# Exploring autoxidation pathways in α-pinene oxidation through particulate measurements

**DOI:** 10.1039/d6ra02412b

**Published:** 2026-07-16

**Authors:** Shuxuan Xu, Zhengjin Li, Pengcheng Zong, Jiao Liu, Song Wang, Xiaojing Shen

**Affiliations:** a Nanjing Meteorological Bureau NO.121, Zhongxindadao, Jianye Nanjing Jiangsu 210019 China xsxnjlh@163.com; b Jiangsu Climate Center, Jiangsu Meteorological Administration No. 8 Yushun Road Jianye District Nanjing Jiangsu 210019 China; c Key Laboratory of Atmospheric Chemistry (LAC), China Meteorological Administration (CMA) No. 46 South Zhongguancun Street Haidian District Beijing 100081 China

## Abstract

In this study, we examine the oxidation pathways of α-pinene and their influence on the formation of highly oxygenated organic molecules (HOMs) and secondary organic aerosols (SOA) using chamber-based particulate measurements combined with COSIMA-SOA and PyCHAM box-model simulations. Two chamber datasets, MAC20120215 and AIDA20081127, were used to test whether observed particle mass and size distributions can be reproduced by three autoxidation-initiation routes: (1) ozone (O_3_) attack on α-pinene, (2) hydroxyl radical (OH) attack on α-pinene, and (3) later-generation oxidation of α-pinene products. Simulations without HOM formation underestimated SOA mass, whereas the inclusion of first-generation and later-generation HOM pathways substantially improved model-measurement agreement. The results indicate that no single pathway alone is sufficient under the investigated conditions and that later-generation HOM chemistry is required to reproduce the second-stage SOA growth observed in AIDA. Although direct HOM measurements were not available, this combined particulate-measurement and modelling approach provides useful constraints for improving simplified mechanisms of organic oxidation and aerosol formation.

## Abstract 2

We investigate α-pinene oxidation pathways, showing that ozone, hydroxyl radicals, and subsequent oxidation processes significantly contribute to highly oxygenated organic molecules (HOMs) and secondary organic aerosols (SOA) formation.

## Graphical abstract

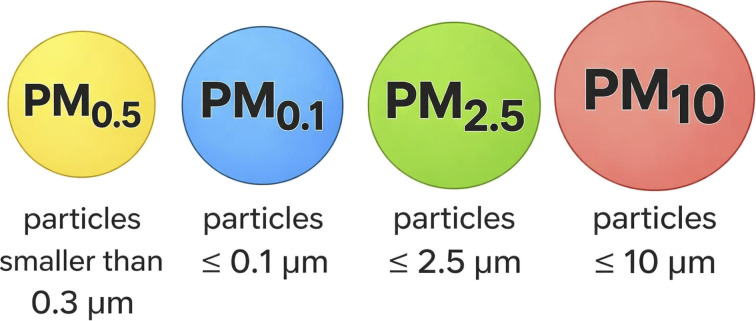

## 1. Introduction

**Fig. 1:**
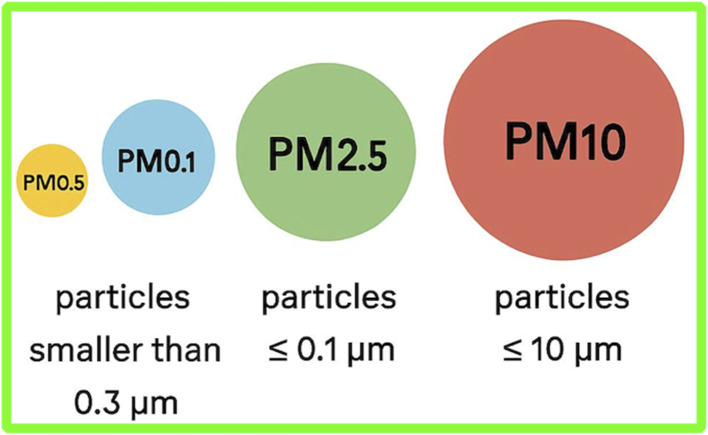
Classification of particulate pollutants by size range.

Particulate matter (PM) consists of airborne solid particles and liquid droplets that vary widely in chemical composition, morphology and size.^1^ Based on particle size, PM is commonly classified as quasi-ultrafine particles (<0.3 µm), ultrafine particulate matter (≤0.1 µm), fine particulate matter (≤2.5 µm) and coarse particulate matter (≤10 µm), respectively (Fig. 1).^1^

Research has shown that the autoxidation of α-pinene oxidation products^2^ leads to the formation of highly oxygenated organic molecules (HOMs), which significantly contribute to particulate matter in the ambient atmosphere through the production of secondary organic particulate matter (SOPM).^3–5^ This process has important implications for climate and air quality.

HOMs are formed through autoxidation, a process involving sequential intramolecular H-shifts followed by oxygen addition to peroxy radicals in the gas phase. This process leads to the formation of compounds containing several oxygen atoms, commonly at least six oxygen atoms. HOMs have been observed after the reaction of α-pinene with ozone, here referred to as first-generation O_3_ oxidation.^6–10^ Autoxidation can also be initiated by hydroxyl radical (OH) attack on α-pinene, here referred to as first-generation OH oxidation.^4,11–15^ Although the production of HOMs from later-generation α-pinene oxidation products remains less well studied, Crounse *et al.*^2^ suggested that autoxidation becomes theoretically more likely as molecules accumulate oxygen-containing functional groups during organic ageing. In addition, Schobesberger *et al.*^3^ and Ye *et al.*^16^ reported molecules with HOM-like characteristics derived from later-generation α-pinene products.

As noted above, several studies have investigated HOM formation from α-pinene through first-generation O_3_ and OH reactions, as well as through later-generation processes. Some of these studies have also examined how individual pathways affect SOPM formation. SOPM is particularly relevant because of its significant impact on climate and air quality.^5,17–20^ Evaluating all three HOM formation pathways is therefore essential, given the variability in oxidant levels and the different degrees of organic ageing occurring in the ambient atmosphere. Although additional pathways may also contribute to HOM production, for example by allowing all peroxy radicals to autoxidize, as suggested by Jenkin *et al.*,^21^ current observational limitations make such a comprehensive approach impractical.

Recent literature has substantially refined the mechanistic understanding of α-pinene oxidation and SOA formation. Quantum-chemical and experimental work has shown that α-pinene ozonolysis can rapidly generate highly oxygenated products because the excess energy released during the initial ozonolysis step can facilitate the formation of peroxy-radical intermediates and rapid oxygen addition.^22^ This supports the inclusion of an O_3_-initiated HOM pathway in simplified mechanisms. At the same time, OH-initiated oxidation and subsequent multi-generational ageing can produce additional oxygenated products and modify the volatility distribution of the aerosol, indicating that first-generation pathways alone may be insufficient to reproduce later-stage SOA growth.^23^ Recent chamber and mechanistic studies have also demonstrated that NO_*x*_ conditions strongly affect RO_2_ fate, highly oxygenated organic nitrate formation, accretion-product formation and total SOA yield;^24^ consequently, mechanisms derived under low-NO_*x*_ chamber conditions should be applied to ambient atmospheres with caution. In addition, comparisons between purified-air and ambient-air chamber matrices show that background pollutants, radical chemistry and seed-particle composition can alter SOA production from α-pinene photooxidation.^25^ Aqueous and particle-phase OH ageing may further change the composition, O ratio and reactivity of α-pinene-derived SOA. Together, these studies show that α-pinene SOA formation reflects the combined effects of O_3_-, OH- and NO_3_/NO_*x*_-related oxidation, multi-generational chemistry, gas-particle partitioning, wall losses and particle-phase processing. Therefore, the present study focuses on testing whether a simplified three-pathway HOM representation can reproduce observed particle mass and size evolution, while explicitly acknowledging that direct HOM measurements and broader NO_*x*_/NO_3_ conditions are required for full atmospheric generalization.^24–26^

Previous studies simulating HOMs have not explicitly incorporated all three formation pathways.^5,16,27,28^ Furthermore, previous modelling studies did not compare simulations with empirical data in a way that clearly distinguished the contribution of each pathway. However, we acknowledge that, in some previous modelling studies, all three pathways may have been implicitly included in the overall HOM yield from α-pinene.^5,16^

The objectives of this study are: (i) to identify the simplest chemical mechanism required to reproduce observed PM; (ii) to extend the Master Chemical Mechanism (MCM)^21,29^ by incorporating the three HOM formation pathways; (iii) to test the different HOM formation pathways, both individually and in combination, against observed PM and determine their relative contributions; and (iv) to assess whether the identified simplified mechanism has the potential to serve as a generally applicable model.

The present study does not aim to provide a complete molecular-level description of HOM chemistry beyond what has already been published. Instead, it focuses on a limited set of chamber conditions and evaluates whether a simplified three-pathway HOM representation can reproduce observed SOPM measurements. The novelty of this work lies in explicitly simulating the three HOM formation pathways together with non-HOM oxidation products and comparing them with particulate measurements. This approach allows the different HOM formation routes to be assessed using particle-based observations and provides useful information for future experimental and modelling studies.

## 2. Methodology

This section introduces the models and outlines the chamber setup. All experiments were repeated three times to ensure data reproducibility.

### 2.1 Experiments

This research investigates the dark ozonolysis of α-pinene using data from two chamber facilities. In the following text, they will be referred to as Manchester aerosol chamber (MAC)20120215 and Aachen institute for advanced study in computational engineering science (AIDA)20081127, respectively. In short, the distinguishing feature between the experiments is that, although an initial injection occurs at the start of both experiments, AIDA20081127 includes a later injection, allowing the OH-driven aging effect to be isolated. For both MAC20120215 and AIDA20081127, measured particle number size distributions were converted into total secondary organic aerosol mass concentrations, assuming an average density of secondary organic aerosols (SOA) of 1.3 g cm^−3^.^30^ The value of 1.3 g cm^−3^ was adopted as a representative average density for α-pinene-derived SOA and to maintain consistency with previous chamber modelling studies. We acknowledge that the effective aerosol density may vary during oxidation and ageing because changes in oxygen content, oligomer or accretion-product formation, water uptake and volatility distribution can modify particle composition. Therefore, the use of a constant density introduces uncertainty into the absolute SOA mass estimates. However, because the same density was applied consistently to all experiments and simulations, this assumption is expected to have limited influence on the relative comparison among the different HOM formation pathways.

#### 2.1.1 MAC20120215

The MAC20120215 campaign was conducted on 15 February 2012 using the MAC, an 18 m3 FEP Teflon chamber (3 m × 3 m × 2 m). Experiments were performed under controlled dark conditions. Complete technical specifications of the MAC facility are available in previous publications.^31,32^

The experiment took place in a dark environment at a steady temperature of 291 K and 65% relative humidity. Initial reactant levels included 30 ppbv α-pinene, 25 ppbv ozone, and 1.8 ppbv NO_2_. Over the four-hour period, we observed nucleation processes without adding seed particles. The chamber's climate control system maintained stable environmental conditions, including temperature, humidity, and pressure. Particle size distributions were measured with a TSI 3080 scanning mobility particle sizer (SMPS). In summary, the MAC20120215 campaign aimed to track changes in the particle number size distribution over time during dark ozonolysis of α-pinene, with sufficient duration to observe multiple generations of oxidation products.

#### 2.1.2 AIDA20081127

The AIDA20081127 experiment was performed on 27 November 2008 in the 84.3 m^3^ AIDA chamber.^32^ This aluminum-cylinder reactor features precise temperature, humidity, and pressure controls within a climate-controlled enclosure, with detailed chamber specifications described in ^33–35^.

This two-phase experiment consisted of:

1. An initial 2.2-hour dark ozonolysis phase with α-pinene (33 ppbv) and excess ozone (550 ppbv) at 293.3 K and 42% RH, ensuring complete α-pinene consumption, followed by.

2. A secondary oxidative aging phase is initiated by continuous injection of 2,3-dimethyl-2-butene (DMB) to generate OH radicals. The second phase was specifically designed to isolate OH-driven aging processes, as detailed in ^30^. The ozone excess (550 ppbv) guaranteed that all α-pinene was consumed prior to DMB introduction at *t* = 2.2 hours.

OH radical production was initiated through DMB ozonolysis to drive the ageing process. At *t* = 1.42 hours after ozonolysis began, 3-pentanol was introduced as an indirect OH tracer. Despite the absence of seed particles, spontaneous nucleation was observed. Particle size distributions were continuously monitored using a TSI 3070 SMPS. We note that the reason the two experiments used different initial conditions is that they had different objectives: the MAC experiment simulated typical atmospheric ozonolysis, whereas the AIDA run deliberately used excess ozone to fully deplete α-pinene before aging. In all cases, however, we applied the same HOM autoxidation mechanism in the model. That both datasets can be fit well with the same mechanism demonstrates its general validity. In our simulations we explicitly account for T, RH, O_3_, NO_*x*_, *etc.*, so the consistent agreement under different conditions further confirms that using a single HOM mechanism is appropriate for both experiments.

Because α-pinene was consumed before the DMB-driven OH-aging phase, the second stage of AIDA20081127 mainly isolates OH-driven processing of first-generation oxidation products. A complete separation is not claimed, because residual oxidants and products may overlap chemically; however, the staged design strongly reduces direct α-pinene ozonolysis during the second phase and allows the role of later-generation OH chemistry to be evaluated.

To improve clarity of the reaction scheme, the main molecular species and structural identifiers used in the model are summarized as follows: α-pinene is a bicyclic monoterpene (C_10_H_16_; SMILES: CC1=CCC2CC1C2(C)C); pinonaldehyde, used here as the surrogate for later-generation oxidation, is an oxygenated α-pinene oxidation product (C_10_H_16_O_2_; commonly represented as a carbonyl-containing ring-opening product). The model species P_1_–P_4_ and HOM-LVOC/HOM-ELVOC terms are not single molecular structures; they are lumped proxy species representing volatility classes and formation pathways. Therefore, their “structure” should be interpreted as a model-defined molecular aggregate or surrogate pool rather than a unique compound.

### 2.2 Simulation setup

This study presents a comparative analysis of experimental results using two distinct box modeling approaches:

The measured chamber pressure was used as an input boundary condition in each simulation, while the pure-component saturation vapor pressures of condensable species were estimated at the corresponding experimental temperature, namely 291 K for MAC20120215 and 293.3 K for AIDA20081127 and then applied in the gas-particle partitioning calculation. The measured temperature, relative humidity, pressure, O_3_, NO_2_/NO_*x*_ and α-pinene concentrations for each experiment were used as model inputs. The measured temperature, relative humidity, pressure, O_3_, NO_2_/NO_*x*_ and α-pinene concentrations for each chamber experiment were used as input parameters in both COSIMA-SOA and PyCHAM. Thus, MAC20120215 was simulated at 291 K and 65% RH, whereas AIDA20081127 was simulated at 293.3 K and 42% RH, consistently with the experimental conditions. These imposed boundary conditions directly influence the simulated oxidation pathways by affecting oxidant availability, reaction rate constants, vapor pressures, gas-particle partitioning, particle growth and wall-loss processes. Therefore, the reported temperature and humidity values were not only used to describe the chamber experiments, but were explicitly imposed in the simulations to constrain the chemical and microphysical evolution of α-pinene-derived HOM and SOA formation.

The MAC20120215 and AIDA20081127 simulations were initialized using their measured chamber conditions as boundary inputs, namely 291 K and 65% RH for MAC and 293.3 K and 42% RH for AIDA, together with the corresponding measured pressure, α-pinene, O_3_ and NO_2_/NO_*x*_ concentrations; these inputs constrained the reaction kinetics, vapor pressure estimates, gas-particle partitioning and wall-loss processes in both modelling frameworks.

1. PyCHAM (Python-based chemistry with aerosol microphysics;^36^ is a recently developed framework, and.

2. The established COSIMA-SOA platform (Computer simulation of aerosol with secondary organic aerosol formation);^33,37^ is systematically evaluated through intercomparison and against chamber experimental data. Both modeling frameworks incorporate the following key features in this analysis:

○ Gas-phase chemistry.

○ Gas-particle partitioning.

○ SOA particles are assumed to be multi-component, ideal mixtures.

○ Gas-wall interaction.

○ Particle-wall interaction.

○ Coagulation.

○ Nucleation.

The Master MCM^29^ serves as the core chemical scheme, with further extensions described below. Neither model includes heterogeneous or particle-phase chemistry; therefore, if these processes significantly affect SOA formation, their impacts will be implicitly incorporated into the fitted parameters of the extended chemical scheme. Gas-particle partitioning is modeled using a kinetic framework in both models in eqn (1).^38^1
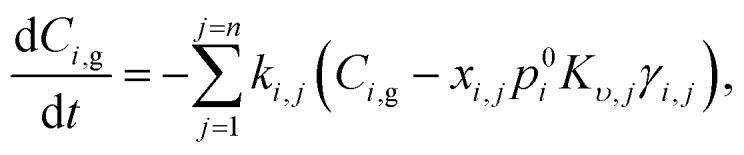
where *i* denotes the component of interest, g represents the gas phase, *C* is concentration, *n* is the number of particle size bins, and *j* refers to a specific particle size bin. *x* is the mole fraction, *p*_0_ is the pure component saturation vapor pressure, *K*ν is the Kelvin factor, and *γ* is the activity coefficient (assumed to be unity). *k* is the mass transfer coefficient, as detailed below. The assumption of unity activity coefficients is supported by both empirical evidence^39^ and structure–activity relationship studies,^40^ indicating that it serves as a reasonable first approximation.

The mass transfer coefficient for gas-particle partitioning follows,^31^ assuming a unity accommodation coefficient (*α* = 1). In reality, if HOMs (which dominate the estimated PM mass in our system) had *α* << 1, SOA formation would be slower due to increased wall deposition losses. However, any non-unity accommodation effects are implicitly accounted for in our fitted wall-loss parameters. This assumption is supported by Krechmer *et al.* (2017), who showed that low-volatility organic compounds generally exhibit *α* ≈ 1.^41^

Neither model considers nucleation first principles. Instead, they fit particle number-size distributions observed during nucleation events. This is done by adding a specific particle mode to the box model over a period that matches the observations.

Models use the same particle-phase component density as in the conversion from particle-number-size-distribution measurements to particle mass concentration (1.3 g cm^−3^). Particle loss to the wall is assumed to be irreversible in both models. Below are descriptions of the setups specific to each model.

#### 2.2.1 COSIMA-SOA

COSIMA-SOA and PyCHAM were used for complementary purposes. COSIMA-SOA was applied as a minimal diagnostic framework to determine whether a small number of HOM-related proxy products could reproduce the observed particle mass evolution. In contrast, PyCHAM was used as a more chemically constrained framework, allowing the same HOM formation pathways to be tested together with MCM-derived condensable products, gas-wall partitioning and aerosol microphysical processes. Therefore, COSIMA-SOA was used to identify the minimum chemical complexity required, whereas PyCHAM was used to evaluate the robustness of the pathway interpretation under a more detailed modelling structure.

It should be noted that eqn (1) describes gas-particle partitioning and not a chemical reaction pathway. In eqn (1), the concentration terms refer to the gas-phase and particle-phase concentrations of a given condensable species, and the equation determines how each species partitions between the gas phase and aerosol particles. The chemical reactions simulated in this study are instead represented by the COSIMA-SOA reactions in eqn (2)–(5) and by the PyCHAM reactions RP_0_–RP_5_. In these schemes, the main reactants are α-pinene, O_3_, OH and pinonaldehyde, while the products are lumped proxy species representing first-generation and later-generation low-volatility or extremely low-volatility oxidation products. Intermediate radical species involved in autoxidation, such as RO_2_ radicals, H-shift intermediates and oxygen-addition products, are not individually resolved, but are represented through these lumped HOM surrogate products.

COSIMA-SOA is described in detail by^33^ and.^37^ COSIMA-SOA uses MCM v3.2 to drive the production and consumption of oxidants and α-pinene; however, MCM components were disallowed from condensing into particles. Only components from the COSIMA-SOA autoxidation extension to MCM were permitted to undergo gas-particle partitioning. This assumption was adopted to keep COSIMA-SOA as a minimal diagnostic framework and to isolate the contribution of the HOM-related extension products to particle growth. We acknowledge that semi-volatile MCM species may also contribute to SOA formation, particularly under conditions of high oxidant exposure or reduced wall losses. Their contribution is therefore evaluated more explicitly in the PyCHAM simulations, where MCM-derived condensable products are included. Consequently, the COSIMA-SOA results should be interpreted as a simplified constraint on the minimum HOM-related chemistry required to reproduce the observed particle mass evolution, rather than as evidence that MCM semi-volatile products are negligible.

The extension, along with the volatilities and gas-wall partitioning rates of the extension components, was generated iteratively to achieve the best agreement with observations while satisfying the two criteria:

(i) including HOMs generated from 1st-generation O_3_ and

(ii) containing the minimum number of reactions and products possible [see eqn (2)–(5)]:

2 *kC*_0_: α-pinene + O_3_ → *n*_1_P_1_ + *n*_2_P_2_ + *n*_3_P_3_

3
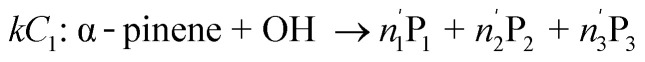

4 *kC*_2_: P_2_ + OH → P_4_

5 *kC*_3_: P_3_ + OH → P_4_

In the above chemical scheme, the oxidation of α-pinene is simulated to yield four product proxies (P_1_–P_4_). The pure component vapor pressures increase in the order P_1_ < P_4_ < P_2_ < P_3_ (Table 1), with P_3_ being sufficiently volatile to make gas-particle and gas-wall partitioning negligible. The proxy species P_1_–P_4_ should not be interpreted as single chemical compounds with unique molecular structures. Rather, they are lumped surrogate species representing classes of α-pinene oxidation products with similar volatility and formation behavior in the model. In this framework, P_1_ represents low-volatility first-generation products, P_2_ represents semi-volatile first-generation products, P_3_ represents more volatile first-generation products, and P_4_ represents lower-volatility later-generation products formed by further OH oxidation of P_2_ and P_3_. Therefore, each proxy species represents a model-defined molecular aggregate or surrogate pool, and the chemical composition within each class may vary. Consequently, no unique molecular structure can be assigned to P_1_–P_4_.

**Table 1:** Parameters of chemical scheme extension components used in COSIMA-SOA and PyCHAM for both experiments^a^

Proxy name	*M*_w_ [g mol^−1^]	*D* [cm^2^ s^−1^]	Volatility [Pa]	Wall loss rate in AIDA [s^−1^]	Wall loss rate in MAC [s^−1^]
**PyCHAM**					
HOM-ELVOC-O_3_	434	0.033	< 1 × 10^−15^	3 × 10^−4^	1.3 × 10^−3^
HOM-LVOC-O_3_	266	0.046	3 × 10^−4^	3 × 10^−4^	1.3 × 10^−3^
HOM-ELVOC-OH	434	0.033	< 1 × 10^−15^	3 × 10^−4^	1.3 × 10^−3^
HOM-LVOC-OH	266	0.046	3 × 10^−4^	3 × 10^−4^	1.3 × 10^−3^
HOM-ELVOC-PINAL	478	0.031	< 1 × 10^−15^	3 × 10^−4^	1.3 × 10^−3^
HOM-LVOC-PINAL	264	0.046	3 × 10^−4^	3 × 10^−4^	1.3 × 10^−3^

**COSIMA**					
P_1_	186	0.049	6 × 10^−6^	1.5 × 10^−4^	1.6 × 10^−4^
P_2_	168	0.049	5 × 10^−4^	2.9 × 10^−4^	1.7 × 10^−4^
P_3_	168	0.049	N/A	N/A	N/A
P_4_	186	0.049	3 × 10^−5^	1.1 × 10^−4^	1.5 × 10^−4^

a P_3_ was remained predominantly in the gas phase, with negligible gas-particle partitioning and wall uptake due to its high volatility.

The reaction of O_3_ and OH (rates from MCM v3.2) with α-pinene leads to the direct formation of product proxies P_1_ (low volatility), P_2_ (semi-volatile), and P_3_ (volatile). The later-generation low-volatility proxy product P_4_ is generated through OH oxidation of P_2_ and P_3_ according to the following reactions: P_2_ + OH → P_4_*k* = 9 × 10^−12^ cm^3^ molecule^−1^ s^−1^; P_3_ + OH → P_4_*k* = 5.5 × 10^−11^ cm^3^ molecule^−1^ s^−1^. These second-order rate coefficients are within the typical range of OH reaction rate constants reported in the Master Chemical Mechanism (MCM v3.2).

The optimum branching ratios (
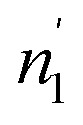
, 
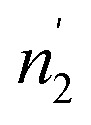
, 
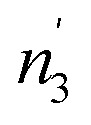
) are only slightly different for the two experiments. For MAC20120215, they are: 0.08, 0.2, 0.1, 0.08, 0.2, 0.1; and for AIDA20081127, they are 0.08, 0.23, 0.135, 0.08, 0.23, 0.13.

In COSIMA-SOA, size-dependent particle wall losses are calculated using the formulations of Naumann (2003).^37^ For gas-phase species, the model treats wall deposition as an irreversible first-order process, with rates constrained by:

(1) An upper limit determined by gas-phase diffusion through the chamber's boundary layer (thickness parameterized following Bunz and Dlugi (1991).^42^

(2) Optimized values for MCM and proxy compounds (Table 1) that achieve model-observation agreement while remaining consistent with Mohr *et al.* (2016) measurements.^43^

#### 2.2.2 PyCHAM

PyCHAM (described fully in O’Meara *et al.* (2021)^36^) treats gas-wall partitioning as a reversible process. However, with the wall's effective absorbing mass concentration set to 1 × 10^3^ g m^−3^ in our implementation, semi-volatile and low-volatility compounds effectively exhibit irreversible wall deposition throughout the simulation period.

The gas-wall partitioning rate constants for HOMs in PyCHAM are listed in Table 1 (MCM components share the same rate constants as HOMs) and align with those reported for another Teflon chamber.^44^ However, wall loss rates differ greatly between chambers (Table 1), with MAC showing losses that are 20 times faster than those in AIDA. This variation matches findings by Kretchemer *et al.* (2009),^33^ who observed that gas-wall partitioning rates in a Teflon chamber^45^ (Pathak *et al.*, 2007) were 20–200 times higher than in the aluminum AIDA chamber under similar experimental conditions.

For MAC20120215, the particle wall-loss rate kernel was fitted to observed size-dependent particle losses,^31^ while for AIDA20081127, the wall-loss kernel was calculated using.^46^ The MCM extension in PyCHAM includes the following simplified HOM formation reactions:RP_0_: α-pinene + O_3_ → *α*HOM-LVOC-O_3_RP_1_: HOM-LVOC-O_3_ + HOM-LVOC-O_3_ → HOM-ELVOC-O_3_RP_2_: α-pinene + OH → *β*HOM-LVOC-OHRP_3_: HOM-LVOC-OH + HOM-LVOC-OH → HOM-ELVOC-OHRP_4_: pinonaldehyde + OH → *γ* HOM-LVOC-PINALRP_5_: HOM-LVOC-PINAL + HOM-LVOC-PINAL → HOM-ELVOC-PINALwhere RP_0_ and RP_2_ are the reaction rate coefficients for α-pinene ozonolysis and oxidation by OH, respectively (using MCM values). Meanwhile, *α* and β are branching ratios for the 1st-generation O_3_ and OH initiation, respectively. Unless stated otherwise below, *α* was set to 0.055 and *β* to 0.01 to broadly align with reported molar yields. For the rate coefficients of accretion (RP_1_, RP_3_, and RP_5_), a value of 5 × 10^−11^ cm^3^ molecule^−1^ s^−1^ was used, following previous established data.^31,46^ RP_4_ denotes the reaction rate coefficient for OH oxidation of pinonaldehyde (using the MCM value), while *γ* represents the branching ratio for later-generation HOM formation. The value of *γ* has been scarcely documented in previous studies; however, previous observations and modelling studies suggest that later-generation oxidation products of α-pinene can contribute substantially to SOPM formation and may be comparable to, or even greater than, first-generation pathways.^3,16^ Accordingly, *γ* was set to 0.06, approximately equal to the sum of *α* and *β*.

Pinonaldehyde was selected as the representative later-generation precursor because it is a major and well-established first-generation oxidation product of α-pinene ozonolysis. In this model, pinonaldehyde is not intended to represent the full complexity of all α-pinene oxidation products, nor is it treated as identical to α-pinene. Rather, it is used as a chemically meaningful surrogate for oxygenated later-generation products that can undergo further OH oxidation and initiate HOM formation. This choice allows the later-generation pathway to be represented in a simplified and traceable way, while remaining consistent with the lumped nature of the PyCHAM mechanism.

Therefore, the kinetic and thermodynamic parameters used here should not be interpreted as unique molecular constants for all α-pinene oxidation products, but rather as observation-constrained parameters for lumped surrogate pathways.

**Fig. 2:**
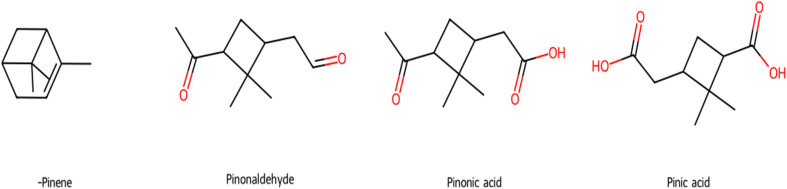
Molecular structures of α-pinene and selected oxidation products discussed in the α-pinene oxidation mechanism. Pinonaldehyde is used in the model as a representative later-generation precursor, while HOM-LVOC and HOM-ELVOC species represent lumped volatility-based surrogate products rather than unique molecular structures.

To further support the interpretation of the simplified chemical scheme, the molecular structures of α-pinene and selected oxidation products are reported in Fig. 2. α-Pinene oxidation is initiated mainly by O_3_ addition to the endocyclic double bond or by OH radical attack. Ozonolysis produces highly reactive Criegee and radical intermediates, which rapidly react with molecular oxygen to form peroxy radicals (RO_2_). These RO_2_ species can undergo intramolecular H-shifts followed by further O_2_ addition, leading to autoxidation and the formation of highly oxygenated organic molecules. In parallel, first-generation products such as pinonaldehyde may undergo further OH oxidation, producing later-generation low-volatility compounds that contribute to HOM and SOA formation. This scheme therefore provides a simplified representation of the main α-pinene oxidation routes relevant to SOA formation, while acknowledging that the lumped HOM-LVOC and HOM-ELVOC species do not correspond to single unique molecular structures.

The Nannoolal method^47,48^ (provided by UManSysProp^49^) was used to estimate the vapor pressures of MCM components. Nannoolal was selected because it has been shown to perform relatively well in comparison with measurements.^50–53^ Several studies have reported a range of HOM volatility.^51,53^ Therefore, this study assumes two types of HOMs. The first represents monomers with an assumed “low volatility” (vapor pressure set at 3 × 10^−4^ Pa). The second corresponds to accretion products and is effectively involatile (vapor pressure set at 1 × 10^−15^ Pa).

Limited empirical data are available on HOMs' vapor pressures under constrained conditions, and the sensitivity of the results to these vapor pressures was not assessed here. However, by setting vapor pressures to represent low volatility (accretion products) and high volatility (monomers), a best estimate is obtained based on the volatility ranges reported in previous studies, indicating that HOMs occupy.^54^

All vapor pressures are pure-component saturation vapor pressures estimated at the experimental temperature used in each simulation. These values enter the kinetic gas-particle partitioning equation through *p*_0_ and determine the tendency of each surrogate species to remain in the gas phase, partition to particles, or be lost to chamber walls.

The carbon number was conserved by assigning 10 carbons to monomers and 20 carbon products to accretion products. The reactions above do not include all possible cross-reactions between HOMs, as this study focuses on evaluating the role of HOMs derived from successive generations of VOC oxidation rather than developing a detailed HOM mechanism. The main goal is to incorporate different oxidation pathways and generational evolution, not to provide an exhaustive view of HOM chemistry.

The PyCHAM chemical scheme extension builds on that of COSIMA-SOA. The scheme used in COSIMA-SOA is less restricted regarding HOM properties but still includes the three main formation pathways. Therefore, COSIMA-SOA offers a ‘minimal working example,’ allowing us to use PyCHAM to explore HOMs in more detail with a more constrained approach.

In COSIMA-SOA, P_1_ follows the same initiation pathways (first-generation oxidation by O_3_ and OH) and has a very low volatility range, like HOM-ELVOC-O_3_ and HOM-ELVOC-O_3_ in PyCHAM. Likewise, P_4_ in COSIMA-SOA represents later-generation, low-volatility products, like the pinon aldehyde HOMs in PyCHAM. Meanwhile, P_3_ corresponds to volatile MCM components (such as pinon aldehyde itself), and P_2_ relates to semi-volatile MCM components in PyCHAM.

### 2.3 Sensitivity analysis

Another important aspect that needs attention is the lack of a systematic sensitivity analysis for key parameters, such as branching ratios, volatility, and contributions from Extremely ELVOC. Although we conducted only two experiments in this study, conducting sensitivity tests is crucial to assess how variations in these parameters could affect the results. Such tests could provide valuable insights into the robustness of our findings and enhance the overall reliability of the model's predictions.

## 3 Results

COSIMA-SOA is used first to investigate the minimum HOMs extension required to match observations from the AIDA and MAC experiments. Then, the PyCHAM HOMs extension is studied, with multiple combinations of HOMs formation pathways used to test their significance in the observed SOPM formation.

### 3.1 Comparison of COSIMA-SOA simulation to observations

**Fig. 3:**
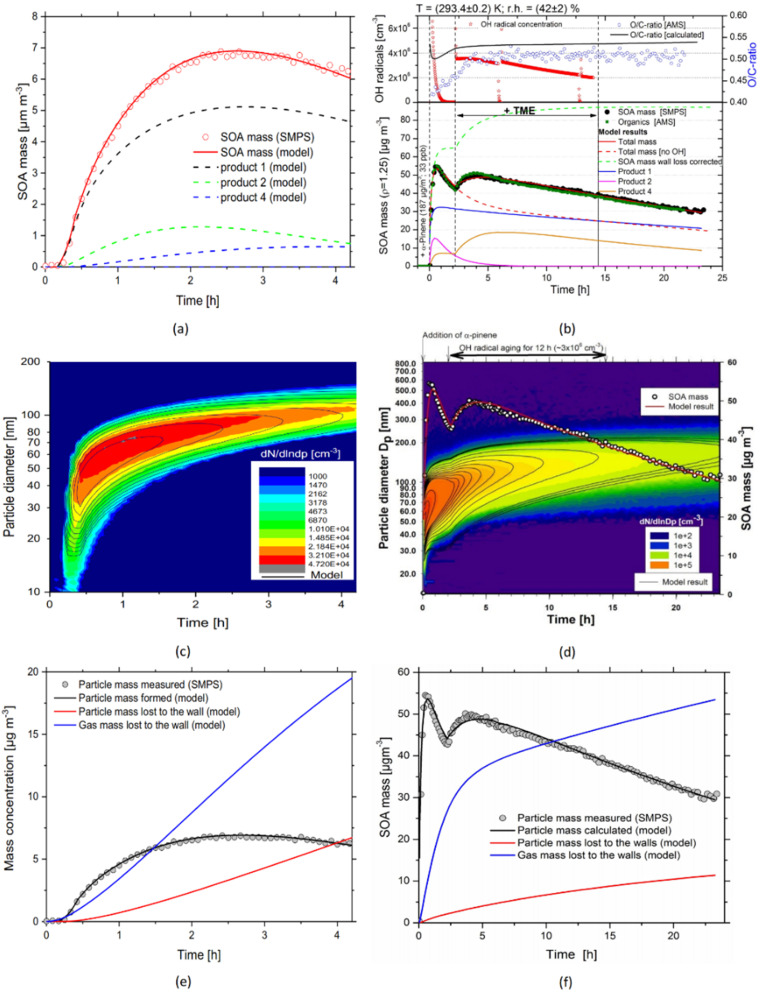
COSIMA simulations compared to observations for (a) MAC20120215 and (b) AIDA20081127. (c) and (d) demonstrate the size distribution modeled by COSIMA for both experiments. (e) and (f) show the measured particle mass compared to the modeled particulate mass and the wall losses *via* particle and gas phase.

Fig. 3 shows the good agreement achieved when the COSIMA-SOA extension parameters were tuned without constraints to achieve the best fit to observations.

Fig. 3a and b show that the proxy component with the lowest vapour pressure, P_1_ (1st-generation oxidation), dominates the particle mass. Furthermore, P_1_ shows little decay in particle-phase concentration compared to P_2_ and P_4_ in both experiments. P_1_ is essential to the reproduction of the early stage of SOPM increase (growth of newly nucleated particles).

P_2_ also represents a significant fraction of SOA, though its particle-phase concentrations decline more rapidly than those of P_1_ (Fig. 3a and b). With a vapour pressure of 5 × 10^−4^ Pa, P_2_ is semi-volatile, leading to evaporation from the particle phase as gas-phase concentrations diminish (due to α-pinene depletion and wall losses). Despite this, Fig. 3a and b demonstrate that P_2_ remains a major SOPM component throughout the MAC20120215 experiment (where α-pinene is not fully consumed) and during the initial phase of AIDA20081127 (prior to α-pinene exhaustion).

Proxy component P_4_ has a volatility lower than P_2_ but higher than P_1_. As a result, its contribution to the secondary organic particulate matter (SOPM) shows an intermediate decay rate (relative to P_1_ and P_2_) in Fig. 3b, indicating partial evaporation from particles as gas-phase P_4_ concentrations decrease. Since P_4_ is produced in later generations, its contribution to particle mass becomes most significant during the second stage of the AIDA20081127 experiment, when OH is generated (Fig. 3b).

In summary, the relatively low volatility proxy components, P_1_ and P_4_, are the main drivers of SOPM formation in the first and second stages of AIDA20081127, respectively, whilst P_1_ is the main SOA component throughout MAC20120215. COSIMA-SOA results, therefore, indicate that the production of both first- and later-generation HOMs is necessary to explain the observed SOPM formation. In the next section, the more-constrained PyCHAM extension is applied to test support of the COSIMA-SOA finding and to better distinguish between HOMs from different 1st-generation oxidation pathways.

### 3.2 Comparison of the PyCHAM simulation without HOMs formation to the observation

For the first test in PyCHAM, HOMs are disallowed so that the components generated by the original MCM scheme are the sole contributors to SOA particle mass production.

**Fig. 4:**
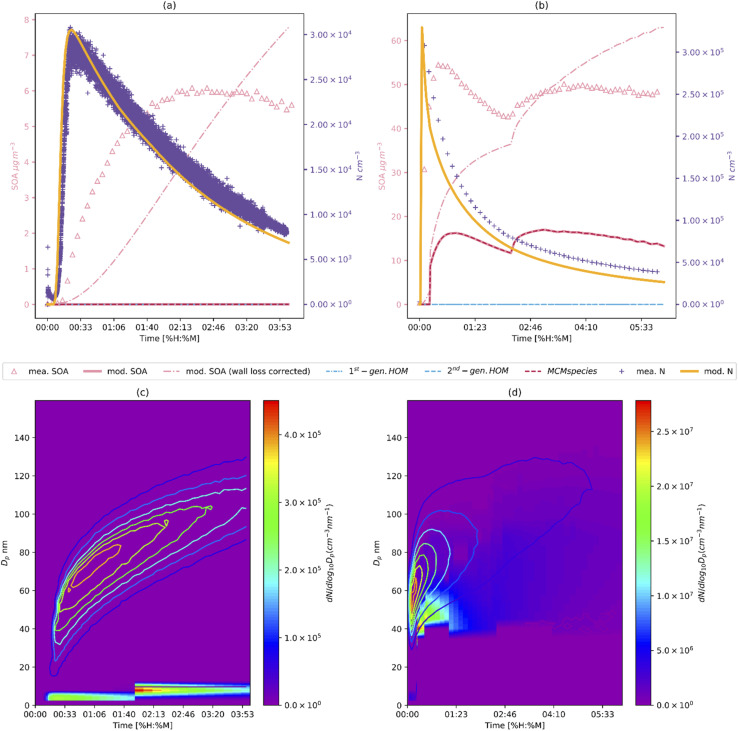
(a) MAC simulation without HOMs formation; (b) AIDA simulation without HOMs formation; (c) the size distribution of the MAC simulation without HOMs formation; (d) the size distribution of the AIDA simulation without HOMs formation.

Fig. 4 demonstrates that the MCM-only components are incapable of reproducing the observed particle mass and number size distributions for both MAC20120215 and AIDA20081127. Fig. 4a, interestingly, shows negligible particle mass production in the MAC20120215 simulation, whilst Fig. 4b shows around 25% of the observed SOPM being produced. Considering eqn (1), the difference in SOA between experiments is due to less gas-phase concentration of vapours: vapour concentrations are enhanced during the early growth stage of AIDA20081127 compared to MAC20120215 due to the greater concentration of ozone in the former (550 *vs.* 25 ppbv).

The simulated SOA temporal profile in Fig. 4b shows trends that reasonably match those observed, with the timing of the SOPM increase fairly well aligned. Consequently, the semi-volatile components generated by the MCM-only chemical scheme appear to be important SOPM contributors. The SOPM contribution of the P_2_ component in COSIMA-SOA (Fig. 3b) supports this idea.

The underestimation of SOPM without HOMs implies a key requirement for lower volatility compounds in the growth of particles, as demonstrated by the contribution of low volatility proxy component P_1_ in Fig. 4a and b above.

### 3.3 Including the HOMs extension in PyCHAM simulations

In the following subsections, we investigate possible formation pathways of HOMs whilst accounting for uncertainty in gas-wall partitioning parameters.

#### 3.3.1 Including 1st-generation HOMs only

**Fig. 5:**
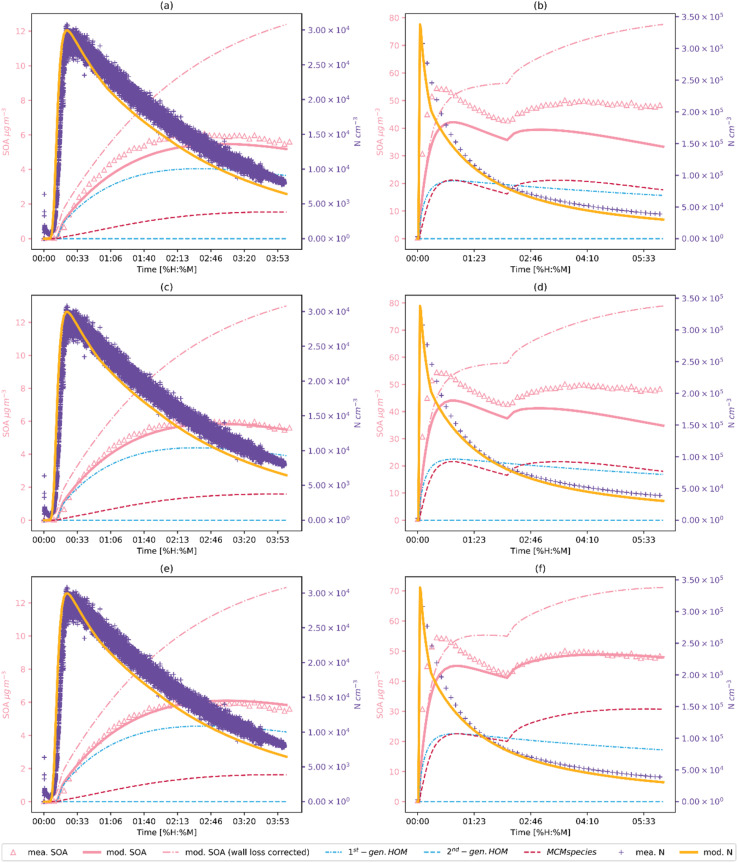
(a) MAC simulation of HOMs formation *via* O_3_ channel only; (b) AIDA simulation of HOMs formation *via* O_3_ channel only; (c) MAC simulation of HOMs formation *via* O_3_ and OH channel (1st-generation only, *C*_w_ = 1 g m^−3^); (d) AIDA simulation of HOMs formation O_3_ and OH channel (1st-generation only, *C*_w_ = 1 g m^−3^); (e) MAC simulation of HOMs formation *via* O_3_ and OH channel (1st-generation only, *C*_w_ = 8 × 10^−4^ g m^−3^); (f) AIDA simulation of HOMs formation O_3_ and OH channel (1st-generation only, *C*_w_ = 5 × 10^−5^ g m^−3^).

Fig. 5 compares the results of MAC20120215 and AIDA20081127 simulations, for the following scenarios: just the 1st-generation O_3_ pathway (Fig. 5a and b); 1st-generation O_3_ and OH pathway (Fig. 5c and d); 1st-generation O_3_ and OH pathway with reduced gas-wall partitioning (Fig. 5e and f).

The purpose of this section is to determine whether both 1st-generation formation pathways are required to best reproduce observations and whether there is an apparent need for SOPM contribution from later-generation HOMs.

Fig. 5a and b show that when HOMs are only formed *via* the O_3_ pathway, the SOPM particle mass loading of both MAC20120215 and AIDA20081127 simulations is lower than the observed. Increasing the molar yield of HOMs *via* the O_3_ pathway does enhance SOA, but we found that a molar yield greater than reported in experimental studies^54–56^ (>10%) is required to reach the observed SOPM.

Fig. 5c and d see an enhancement of SOPM particle mass loading by around 10% compared to Fig. 5a and b, and therefore, improved model-observation agreement, indicating that 1st-generation O_3_ and OH HOMs are necessary to explain the observed SOPM.

While the agreement between the modeled and the observed MAC20120215 experiment is quite good in Fig. 5c, there is a relatively large discrepancy in the AIDA20081127 experiment (Fig. 5d). Note that the excess simulated decay of PM in the second stage (after 2 hours) of AIDA20081127 follows α-pinene exhaustion, therefore, the resolution is not possible through modification of the molar yields of 1st-generation HOMs.

Next, we examine whether agreement can be improved, particularly in the second stage of AIDA20081127, by reducing the competitive sink from the chamber wall. We reduce the wall's effective absorbing mass to a level that substantially suppresses MCM-component condensation on the wall, thereby minimizing particle evaporative losses.

The effective absorbing wall mass concentrations are reduced to 8 × 10^–4^ g m^−3^ and 5 × 10^–5^ g m^−3^ in MAC and AIDA, respectively. Fig. 5f shows a notable model improvement in the second phase of AIDA20081127 due to an increased contribution from MCM components: the boost in OH drives the production of semi-volatile oxidized MCM components, which more readily condense and remain on particles because of their relatively low competitive uptake onto walls. However, an effective absorbing mass of the wall on the order of 1 × 10–5 g m^−3^ is orders of magnitude lower than values reported in chamber gas-wall partitioning and vapor-wall-deposition studies.^33,57–60^ Therefore, we conclude that the reduced effective absorbing mass in the wall scenario is unlikely, and in the following sections, we will test whether later-generation HOMs can better reproduce observed PM.

#### 3.3.2 Including later-generation HOMs only

In the previous section (Fig. 5f), we demonstrated that an OH-driven source of condensable material is required to correctly simulate the second phase of AIDA20081127 (when DMB has been injected to raise OH concentrations and α-pinene has been consumed). With this basis, next, we investigate the potential for PM formation by HOMs from later-generation OH reactions.

**Fig. 6:**
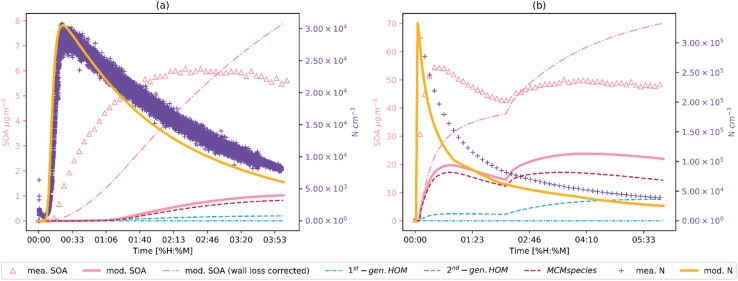
(a) MAC simulation with 2nd-generation HOMs only; (b) AIDA simulation with 2nd-generation HOMs only.

In Fig. 6a and b, the 1st-generation HOM pathways (O_3_ and OH) are disallowed to test whether later-generation HOMs can reproduce PM at all times throughout the experiments. Fig. 6 shows a considerable underestimation of simulated PM, but later-generation HOMs, in combination with MCM components, can reproduce the observed magnitude of PM increase followed by decay in the second phase of AIDA20081127. Any further increase in simulated later-generation HOMs production would overestimate PM change in this second phase; therefore, Fig. 6 shows that later-generation HOMs alone cannot reproduce the total observed PM in either experiment. Combining the findings from Fig. 5 and 6 indicates that all three HOM formation pathways are necessary to achieve the best agreement with observations.

#### 3.3.3 Including HOMs from 1st-generation O_3_ and OH and later-generation

**Fig. 7:**
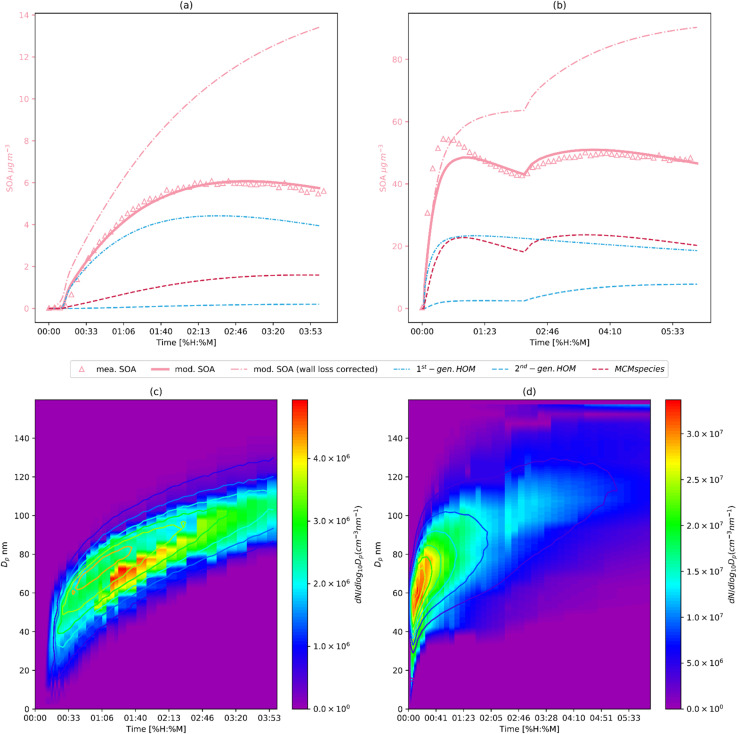
The best simulated cases of MAC20120215 (a and c) and AIDA20081127 (b and d).

In this section, HOMs can form *via* OH-driven later-generation reactions, in addition to 1st-generation HOMs from O_3_ and OH. The results are shown in Fig. 7, which demonstrates the best agreement with observations of all PyCHAM simulations.

**Fig. 8:**
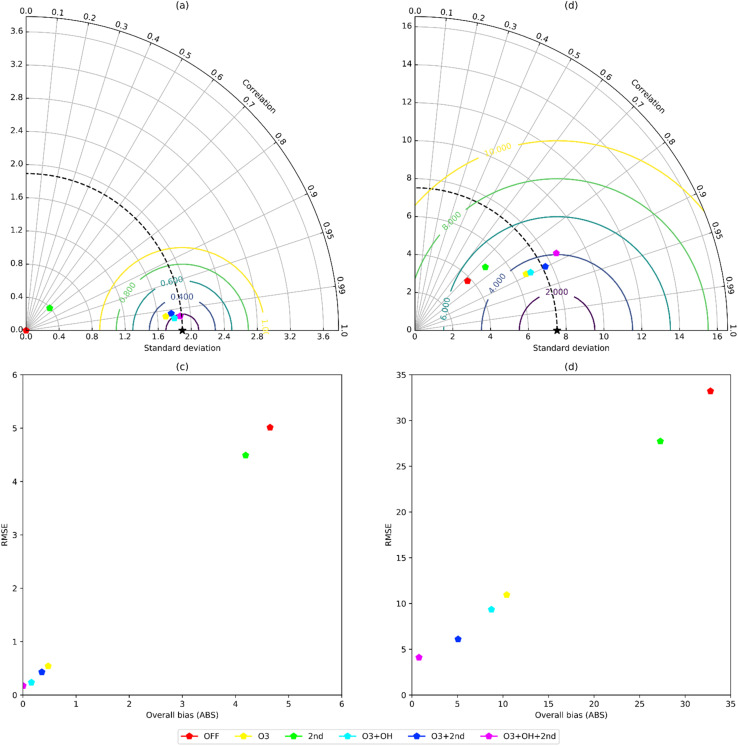
(a) Taylor diagram of all MAC simulations (see main text for plot introduction); (b) Taylor diagram of all AIDA simulations (see main text for plot introduction); (c) classic root mean square error and overall absolute bias of all MAC simulations when compared against measurement; (d) classic root mean square error and overall absolute bias of all AIDA simulations when compared against measurement; *: measurement. Note: red: oFF: no HOM species. Yellow: O_3_: only 1st-generation HOM species from O_3_ pathway. Green: 2nd: only 2nd-generation HOM species from both O_3_ and OH pathways. Cyan: O_3_ + OH: 1st-generation HOM species from both O_3_ and OH pathways. Blue: O_3_ + 2nd: 1st- and 2nd generation HOM species from only O_3_ pathway. Pink: O_3_ + OH + 2nd: 1st- and 2nd-generation HOM species from both O3 and OH pathways (all HOM species).

For MAC20120215 and AIDA20081127, Fig. 7 shows good agreement between simulated and measured PM, whilst Fig. 8 shows that this ‘all HOMs’ model setup gives the lowest root mean square error of all simulations. For MAC20120215 and AIDA20081127, Fig. 7 shows good agreement between simulated and measured PM, whilst Fig. 8 shows that this “all HOMs” model setup gives the lowest root mean square error of all simulations. The improved agreement obtained when the pinonaldehyde-based later-generation pathway is included therefore indicates the need for an OH-driven source of low-volatility material after the first α-pinene oxidation step. It does not uniquely identify pinonaldehyde as the only precursor responsible for this material. Other oxygenated α-pinene products with comparable reactivity and volatility may also contribute under atmospheric conditions and should be resolved in future studies using direct HOM and intermediate-product measurements. It is important to note that Fig. 7b, discrepancy mainly occurs during the initial nucleation and rapid early-growth stage, which is widely recognized as one of the most challenging phases to reproduce quantitatively in chamber SOA simulations.^51,55,56^ In the AIDA20081127 experiment, no seed aerosol was introduced, and particle formation occurred through spontaneous nucleation. In PyCHAM, nucleation is represented using an observation-constrained particle injection approach rather than a fully first-principles nucleation mechanism. Under such transient conditions, small uncertainties in the timing, number concentration, or size distribution of newly formed particles can substantially influence the initial SOA mass accumulation rate.

Second, both PyCHAM and COSIMA-SOA currently neglect heterogeneous and particle-phase chemistry. Experimental and theoretical studies have shown that, following HOM condensation, rapid accretion reactions, including dimerisation and oligomer formation, can decrease volatility and enhance SOA growth.^13,51,55^ Since the present model framework only considers gas-phase production followed by kinetic partitioning, these additional particle-phase mass-enhancing processes are not represented, which likely contributes to the underestimation during the initial stage. Importantly, despite these early-stage discrepancies, the inclusion of later-generation HOM chemistry substantially improves the overall SOA growth trend, aerosol mass evolution, and later-stage agreement compared with simulations without HOM chemistry. Therefore, the main conclusions regarding the importance of later-generation HOM formation remain robust.

Fig. 7c and d show the particle number size distributions for both ‘all HOM’ simulations. The simulated particle-size evolution is displayed as a mesh plot, while the measured distribution is shown as a contour plot. For the MAC20120215 case, the simulation captures the overall envelope of the SMPS-measured distribution well. However, the red band (the highest d*N*/d log *D*_p_) appears shifted to later times, suggesting a delay in modeled particle growth. In contrast, the AIDA20081127 simulation exhibits good agreement between the simulated and observed contours.

Fig. 8a and b show all combinations of HOMs simulation presented above in the form of a Taylor diagram. A Taylor diagram is set in polar coordinates, with the polar axis representing the standard deviation and the angular coordinate representing Pearson's correlation coefficient. The coloured contour lines represent the centred pattern RMS difference.^61^ Except for the cases of ‘no HOM’ and ‘later-generation HOMs only’, which both show a relatively large deviation from the measurement, other combinations of HOMs are relatively close to each other, indicating a similar degree of agreement with measurements.

Instead of the ‘classic’ RMS error, the Taylor diagram uses the centred pattern RMS error, which removes the mean bias between two datasets and instead quantifies the difference in the variation of coincident points. Fig. 8c and d demonstrate the classic RMS error *vs.* overall bias (absolute value), which serves as a general measure of the model-measurement agreement across different HOM scenarios. The distance between the colourized pentagons and the origin denotes the general deviation of each case from the measurement. It is clear in Fig. 8c and d, in both MAC20120215 and AIDA20081127, the ‘all HOM’ simulation gives the best agreement.

### 3.4 Comparison of COSIMA-SOA and PyCHAM simulations

Comparing COSIMA-SOA's ‘minimal working example’ chemical scheme with PyCHAM's better-constrained scheme helps summarize the role of multiple chemical components in PyCHAM's SOA formation.

P_1_ in COSIMA-SOA follows the temporal trend of the 1st-generation HOMs in PyCHAM; likewise, P_4_ replicates the role of later-generation HOMs. The role of P_3_ in COSIMA-SOA is comparable to pinonaldehyde in PyCHAM and, more generally, any later-generation organic capable of initiating HOMs formation. P_2_ does not map well onto any of the PyCHAM components. The relatively quick decay of P_2_ in COSIMA-SOA results is closest in trend to the MCM components in PyCHAM.

However, P_2_ makes no contribution to PM in the second stage of AIDA20081127, whilst MCM components make a substantial contribution. Therefore, P_2_ appears to represent a subset of condensable early-generation MCM components, whilst the MCM component contribution in PyCHAM in the second phase of AIDA20081127 is replicated by P_4_ in COSIMA-SOA. In addition, P_4_ reproduces the role of later-generation HOMs.

For MAC20120215, the modeled particle number-size distributions from COSIMA-SOA and PyCHAM both show some disagreement with observations. Whilst COSIMA-SOA simulates a peak number concentration that is at larger particle sizes than measured (Fig. 3c and d), PyCHAM simulates a peak number concentration at smaller particle sizes than measured (Fig. 8c). Whereas, for AIDA20081127, neither model shows a notable deviation from measured particle number size distributions. Since the PyCHAM chamber is Teflon, and multiple effects have been found to affect particle loss to the wall,^31^ the MAC20120215 discrepancy is not surprising. The fact that the total SOA mass is well reproduced by both models in MAC20120215 indicates that the particle-loss size function is skewed in both models. We do not consider this inaccuracy to compromise our findings on the relative importance of different HOMs formation pathways.

Overall, these results are consistent with previous literature showing that first-generation HOMs are important for initial particle growth, while later-generation oxidation and particle-phase processing can further enhance SOA mass. The improved model-measurement agreement after inclusion of later-generation HOMs is also consistent with recent mechanistic, chamber and OH-aging studies indicating rapid autoxidation, NO_*x*_-dependent changes in HOM composition, and additional low-volatility product formation during α-pinene oxidation.^22–26^

## 4. Discussion

The obtained results are broadly consistent with previous experimental, chamber-based and modelling studies on α-pinene oxidation and SOA formation. Earlier studies demonstrated that O_3_-initiated α-pinene oxidation can rapidly generate highly oxygenated organic molecules through autoxidation processes, contributing to early particle formation and growth.^8,9,55,56^ The importance of OH-initiated oxidation and RO_2_ chemistry is also supported by previous studies showing that OH reactions with α-pinene and its oxidation products can generate low-volatility compounds and accretion products relevant to SOA formation.^13–15^ In addition, later-generation oxidation products have been shown to contribute substantially to secondary organic aerosol production, supporting the inclusion of a later-generation HOM pathway in the present model.^16,17^ The finding that the best model-measurement agreement is obtained only when O_3_-initiated, OH-initiated and later-generation HOM pathways are simultaneously included is therefore in agreement with the available literature, which highlights the combined role of first-generation oxidation, multi-generational ageing, RO_2_ chemistry, gas-particle partitioning and particle-phase processing in α-pinene-derived SOA formation.^22–26^

### 4.1 Limitations of the current HOM formation model

This study employs model simulations of MAC and AIDA reactors under α-Pinene oxidation conditions to investigate HOM formation pathways and SOA evolution. While the simulations successfully reproduced particle number concentrations and size distributions, an important limitation is the lack of direct measurements of HOMs and intermediate products, which weakens validation of the modeled first-generation HOMs, later-generation HOMs, and MCM-derived species.^51,52^ Although the inclusion of the pinonaldehyde-based later-generation pathway improved the agreement between simulated and observed SOA mass, this result should not be interpreted as a molecularly specific attribution to pinonaldehyde alone. In the present lumped modelling framework, pinonaldehyde was selected as a representative oxygenated first-generation product of α-pinene ozonolysis because it is well established in α-pinene oxidation chemistry and can undergo further OH oxidation. Therefore, its role in the model is to represent a broader class of oxygenated α-pinene oxidation products that may act as precursors of later-generation HOMs and low-volatility material. Other compounds formed during α-pinene oxidation, including carbonyls, hydroxycarbonyls, organic peroxides, and multifunctional products with similar OH reactivity and volatility, may also contribute to this pathway. Future studies should therefore combine chamber particle measurements with direct HOM detection,^52^ for example using nitrate chemical-ionization mass spectrometry or comparable real-time high-resolution techniques, together with measurements of key intermediates such as pinonaldehyde and other oxygenated C10 products. Another limitation of the current model is the simplified treatment of HOM-RO_2_ chemistry, where HOM-RO_2_ radicals were assumed to undergo only self-reactions without considering cross-reactions, potentially leading to underestimation of ELVOC formation.^51,55^ The model also does not distinguish between HOM-RO_2_ radicals and stable products, which may affect the accuracy of aerosol formation predictions and atmospheric chemical interpretations. Furthermore, the ELVOC volatility was set to 10^−15^ Pa, substantially lower than the minimum literature value of 2.9 × 10^−13^ Pa, potentially overestimating the contribution of ELVOC to aerosol mass.^51–53^ Nevertheless, sensitivity analysis indicated that ELVOC behaves as fully condensed when volatility remains below 10^−12^ Pa. Future work should therefore explore broader volatility ranges and more detailed HOM-RO_2_ reaction mechanisms, including cross-reactions, to improve predictive accuracy and better represent aerosol formation processes under atmospheric conditions.

In addition, quantum chemical modelling could provide valuable support for evaluating the relative importance of the different α-pinene oxidation pathways. Density functional theory calculations of reaction energy barriers, transition states, H-shift feasibility, O_2_-addition steps and product stability would help identify the most favorable autoxidation routes and clarify whether the simplified pathways represented in the model are kinetically and thermodynamically plausible. Such calculations should therefore be considered in future work to strengthen the mechanistic interpretation of α-pinene-derived HOM and SOA formation. In addition, pathway-specific kinetic and thermodynamic data are required to more rigorously evaluate the relative significance of each α-pinene oxidation route. Although the present model uses reaction rate coefficients from the Master Chemical Mechanism and volatility estimates derived from established structure–activity methods, the simplified HOM scheme does not provide a complete molecular-level description of reaction barriers, transition states, RO_2_ stability, H-shift feasibility, O_2_-addition steps or accretion-product formation. Quantum chemical modelling, including density functional theory calculations of reaction energy profiles and product stability, would therefore provide valuable support for identifying the most favorable oxidation pathways and assessing whether the simplified pathways represented in the model are kinetically and thermodynamically plausible. Such calculations, combined with direct HOM and intermediate-product measurements, should be considered in future work to strengthen the mechanistic interpretation of α-pinene-derived HOM and SOA formation.

### 4.2 Limitations in experimental design and quantification of HOM formation pathways

A notable limitation of this study is the limited representativeness of the experimental conditions. Only two chamber experiments were conducted under extremely low NO_*x*_ conditions, without variations in NO_*x*_ levels or consideration of NO_3_ radical oxidation, limiting the ability to capture the diversity and complexity of real atmospheric environments. Since NO_*x*_ strongly influences RO_2_ chemistry and HOM formation pathways,^24^ future studies should incorporate a broader range of atmospheric conditions, including varying NO_*x*_ concentrations and NO_3_-driven oxidation chemistry, to better reflect realistic atmospheric processes. In addition, although the study qualitatively demonstrates the importance of all three HOM formation pathways, it does not quantitatively determine their relative contributions to SOA formation. The inclusion of pathway-specific contribution percentages or comparative charts would provide clearer insight into the significance of each pathway and strengthen the overall interpretation of the HOM reaction mechanism.^61^

The absence of direct HOM measurements and intermediate species limits the robustness of the mechanistic conclusions and highlights the need for future studies incorporating real-time HOM and intermediate product measurements to validate model predictions and strengthen the atmospheric relevance of the findings.^51,52^ This study employs model simulations of two distinct reactor types (MAC and AIDA) under experimental conditions involving alpha-pinene oxidation. While model simulations suggest potential processes and pathways that can effectively reproduce measurements of particle number, concentration, and size distribution, a critical limitation is the lack of actual measurements of HOMs and intermediate products. Moreover, the lack of rigorous validation of the model against real HOM measurements undermines the robustness of the major claims in this paper. Although the conclusion states that these are “carefully designed experiments”, the absence of empirical data to support the simulations of the three products/groups (1st gen. HOM, 2nd gen. HOM, and MCM species) is a significant gap. Future research should prioritize obtaining actual measurements of HOMs to validate the model and strengthen the conclusions drawn from this work.^51,52^

### 4.3 Relevance of SOA concentrations in the AIDA experiment

Although the study demonstrates that all three HOM formation pathways contribute significantly to SOA formation, the atmospheric relevance of the AIDA chamber experiments should be interpreted with caution. The SOA concentrations observed in the AIDA experiments (∼50 µg m^−3^) are substantially higher than the levels generally represented in ambient regional and global aerosol modelling studies.^17–20^ Such elevated concentrations and different oxidant regimes can modify RO_2_ fate, organic nitrate formation and SOA yields, especially when NO_*x*_/NO_3_ chemistry is important.^24,51,62^ Therefore, the primary objective of this work is not to directly replicate typical atmospheric conditions, but rather to elucidate the underlying reaction mechanisms governing HOM formation from α-pinene oxidation.^45,51^ Furthermore, key HOM formation parameters were derived from only two chamber experiments, despite HOM and SOA production being highly sensitive to precursor concentrations, oxidant levels, and NO_*x*_ conditions. Importantly, NO chemistry, which strongly influences RO_2_ fate and HOM formation, was not incorporated into the chamber setup, limiting the broader applicability of the model parameterizations to ambient atmospheres and other chamber studies.^24,25,62^ In addition, while this study qualitatively establishes the importance of first-generation O_3_ oxidation, OH oxidation, and later-generation pathways, it does not quantitatively determine their relative contributions to SOA formation. Future studies should therefore focus on both quantitative pathway attribution and validation under more representative atmospheric conditions to strengthen the atmospheric applicability of HOM and SOA formation mechanisms.^24,25,62^

### 4.4 Impact of NO_3_ radicals on HOM and SOA formation

In response to the potential role of NO_3_ radicals, it is important to note that NO_3_-initiated oxidation can influence α-pinene oxidation, organic nitrate formation and SOA production, particularly under mixed biogenic-anthropogenic conditions. However, the present study did not directly assess the contribution of NO_3_ radicals. Future studies should therefore incorporate NO_3_-driven pathways into both experimental and modelling frameworks to better evaluate their influence on HOM and SOA formation under atmospherically relevant conditions, particularly because NO_*x*_ and NO_3_ chemistry can substantially modify RO_2_ fate, HOM-ON formation, accretion products and SOA yields.^24,62^ In addition, future work should validate these findings under more representative ambient conditions to strengthen the applicability of chamber-derived conclusions.^25,62^

This study represents the first integrated evaluation of all reported HOM formation pathways, including first-generation O_3_ oxidation, OH oxidation, and later-generation pathways, in relation to particulate matter (PM) observations. The results demonstrate that HOMs from all three pathways contribute significantly to PM formation, consistent with previous studies focusing on individual mechanisms. Combined particulate measurements and mechanistic insights therefore provide an important assessment of the atmospheric relevance of these pathways. Nevertheless, chamber conditions differ substantially from the ambient atmosphere, particularly because α-pinene concentrations in chamber studies can be higher than outdoor levels.^63,64^ Furthermore, chamber walls act as strong condensation sinks that can alter gas-particle partitioning, vapour loss and SOA-yield interpretation in chamber experiments.^33,41,44,46,57–60^ Additional uncertainties arise from HOM branching ratios, vapor pressure estimations, gas-wall partitioning effects, and the use of the Nannoolal method, which may underestimate volatility and thereby influence PyCHAM predictions of MCM-derived PM contributions.^47–50^

### 4.5 Future research directions

Despite recent advances in understanding highly oxygenated molecules (HOMs), correcting experimental artifacts and reducing uncertainties remain ongoing challenges in atmospheric aerosol research. Previous model-outdoor observation comparisons, such as those reported by Roldin *et al.*^17^ and Weber *et al.*^5^ suggest that first-generation HOMs are essential for explaining the observed secondary organic particulate matter (SOPM) formed near α-Pinene emission sources, contributing approximately 20% of particulate matter (PM) mass under real atmospheric conditions. In addition, the role of multi-generational HOMs in ambient aerosol formation is increasingly recognized and warrants further investigation, as supported by recent evidence on rapid α-pinene autoxidation, terpene autoxidation products, and OH-driven aging of α-pinene SOA.^22,23,26^ To deepen our understanding of HOM chemistry and its atmospheric implications, future research should focus on evaluating later-generation HOMs within atmospheric models and validating these predictions against field observations collected near α-pinene emission regions. Furthermore, enhanced atmospheric modeling frameworks are needed to more accurately represent the complex oxidation pathways, chemical interactions, and formation mechanisms governing HOM evolution under realistic environmental conditions.

A major limitation of the present study is the absence of quantitative attribution of each oxidation pathway to secondary organic aerosol (SOA) formation. Specifically, the relative contributions of pathways such as MCM-derived products, O_3_-generated HOMs, OH-generated HOMs, and ELVOC species were not quantified. The inclusion of pathway-specific percentage contributions or comparative visualization, such as contribution charts, would substantially improve the clarity and interpretation of the results by providing a clearer understanding of how individual oxidation pathways influence overall SOA formation and atmospheric PM production. Such quantitative assessments are essential for accurately evaluating the atmospheric significance of different HOM formation routes and for improving predictive aerosol models.

## 5 Conclusions

This study demonstrates that HOM formation from α-pinene oxidation is necessary to reproduce the observed particle-mass evolution in the MAC and AIDA chamber experiments. Simulations performed without HOM chemistry substantially underestimated SOA formation, whereas the inclusion of O_3_-initiated, OH-initiated and later-generation HOM pathways markedly improved the agreement with measured particle mass and size distributions. The results indicate that the O_3_-initiated pathway is particularly important for early particle growth, while OH chemistry provides an additional source of condensable material. Later-generation oxidation products appear to play a key role in sustaining the second-stage SOA growth observed in the AIDA experiment.

Because direct measurements of HOMs and intermediate oxidation products were not available, the proposed mechanism should be interpreted as a simplified representation constrained by particle measurements rather than as a complete molecular description of α-pinene oxidation chemistry. The model results support the need to include O_3_-initiated, OH-initiated and later-generation sources of low-volatility material to reproduce the observed SOA evolution, but they do not allow a unique molecular attribution of all HOM products. Future studies should combine chamber particle measurements with direct HOM and intermediate-product detection, pathway-specific kinetic and thermodynamic data, systematic sensitivity analyses, and experiments under broader NO_*x*_/NO_3_ conditions. Such integrated approaches would help quantify the relative contribution of individual oxidation pathways and improve the atmospheric applicability of α-pinene-derived HOM and SOA formation mechanisms.

## Author contributions

S. X. led the manuscript preparation and data analysis. Z. L. enhanced the NPF module of PyCHAM. P. Z., J. L., S. W., and X. S. contributed to data analysis and manuscript improvement.

## Conflicts of interest

The authors declare they have no conflicts of interest.

## Abbreviations

HOMs: Highly oxygenated organic molecules
SOA: Secondary organic aerosols
PM: Particulate matter
O_3_: Ozone
OH: Hydroxyl radical
SOPM: Secondary organic particulate matter
MCM: Master chemical mechanism
ELVOC: Extremely low volatile organic compounds
LVOC: Low volatile organic compounds
MAC: Manchester aerosol chamber
AIDA: Aachen institute for advanced study in computational engineering science
DMB: 2,3-Dimethyl-2-butene
SMPS: Scanning mobility particle sizer

## Data Availability

Data for this study are available from the corresponding author upon reasonable request.
